# Supplementary material for: Janus particle-engineered structural lipiodol droplets for arterial embolization
Source: Nat Commun. 2023 Sep 11;14:5575. doi: 10.1038/s41467-023-41322-6 (PMC10495453; doi:10.1038/s41467-023-41322-6)
Supplement: Supplementary file 1 — Supplementary Information [file 41467_2023_41322_MOESM1_ESM.pdf]

Supplementary Information

Janus Particle-Engineered Structural Lipiodol Droplets for Arterial Embolization

Sijian Tao[1,2,7], Bingquan Lin[3,7], Houwang Zhou[1], Suinan Sha[1], Xiangrong Hao[1], Xuejiao Wang[1], Jianping Chen[1], Yangning Zhang[1], Jiahao Pan[1], Jiabin Xu[4], Junling Zeng[5], Ying Wang[1], Xiaofeng He[4], Jiahao Huang[2,6]*, Wei Zhao[4]* & Jun-Bing Fan[1]*

[1]Cancer Research Institute, School of Basic Medical Sciences, Southern Medical University, 510515 Guangzhou, P. R. China

[2]School of Biomedical Engineering, Southern Medical University, 510515 Guangzhou, P. R. China

[3]Department of Medical Imaging Center, Nanfang Hospital, Southern Medical University, 510515 Guangzhou, P. R. China

[4]Division of Vascular and Interventional Radiology, Department of General Surgery, Nanfang Hospital, Southern Medical University, 510515 Guangzhou, P. R. China

[5]Laboratory Animal Research Center of Nanfang Hospital, Southern Medical University, 510515 Guangzhou, P. R. China

[6]Department of Critical Care Medicine, Affiliated Hospital of Guangdong Medical University, 524000 Zhanjiang, P. R. China

[7]These authors contributed equally

Correspondence and requests for materials should be addressed to J. F. (fjb2012@mail.ipc.ac.cn); W. Z. (pummpa@smu.edu.cn); J. H. (jhuangaf@connect.ust.hk).

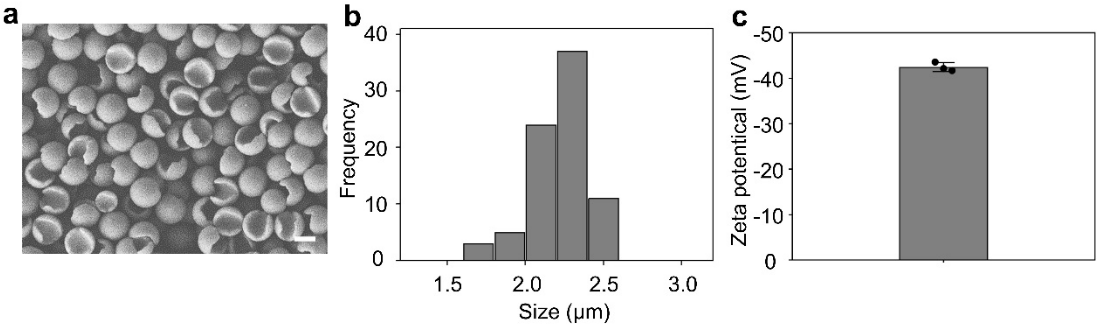

Supplementary Fig 1. Fabrication and characterization of Janus particles. (a) SEM image of Janus particles. Scale bars: 2 μm. (b) Size distributions of Janus particles. (c) Surface potential of Janus particles. n = 3 independent experiments. Data are presented as means ± SD. Experiments were performed three times, with similar results.

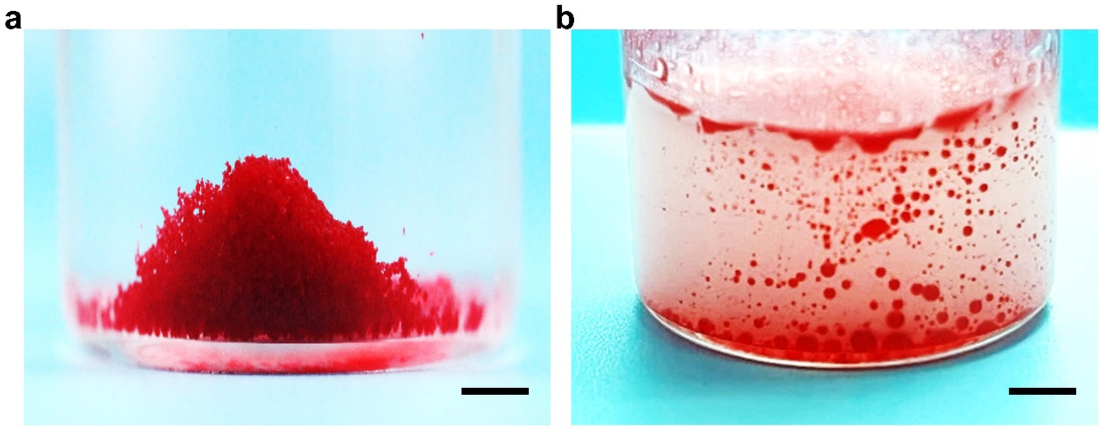

Supplementary Fig 2. The comparison of the fabrication of lipiodol droplets by using poly(styrene-co-divinyl benzene)-poly(acrylic acid) (PSDVB-PAA) Janus particles and spherical poly(styrene-acrylic acid) (PS-PAA) particles. (a) The fabrication of lipiodol droplets by using PSDVB-PAA Janus particles. Scale bar: 1 cm. (b) The fabrication of lipiodol droplets by using PS-PAA particles. When 0.1 mL of lipiodol was mixed with 6 mL of Janus particle (1.5 mg/mL) aqueous solution under shearing, the stable Janus particle-engineered structural lipiodol droplets could be formed. By contrast, spherical PS-PAA particles are difficult to fabricate stable lipiodol droplets. Scale bar: 1 cm. Experiments were performed three times, with similar results.

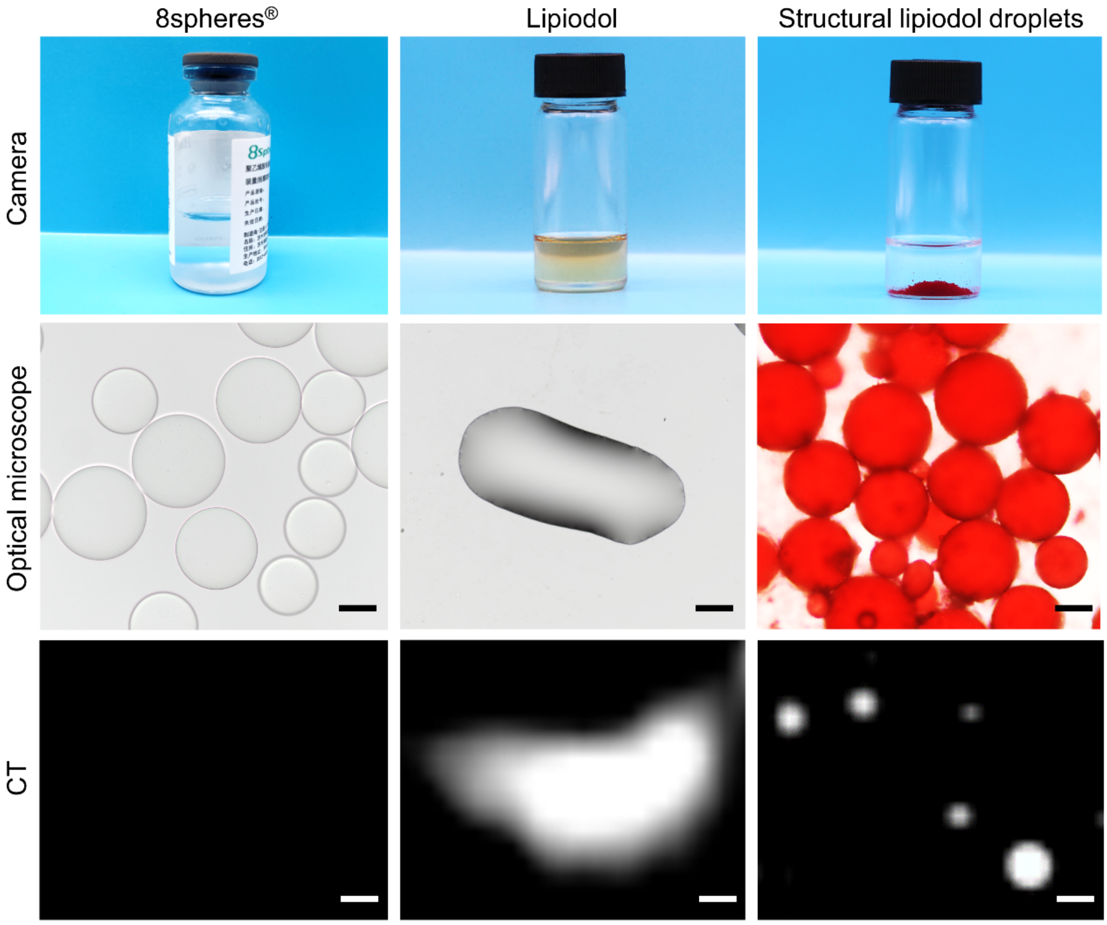

Supplementary Fig 3. Characterization of Janus particle-engineered structural lipiodol droplets, clinical 8spheres® beads and lipiodol. Optical microscope images showed the Janus particle-engineered structural lipiodol droplets and clinical 8spheres® beads were spherical shapes, while lipiodol was a liquid. CT images confirmed the superior radiography capacity of the obtained Janus particle-engineered structural lipiodol droplets and lipiodol, while clinical 8spheres® beads had no radiography capacity. Optical microscope images, Scale bars: 50 μm; CT images, scale bars: 200 μm. Experiments were performed three times, with similar results.

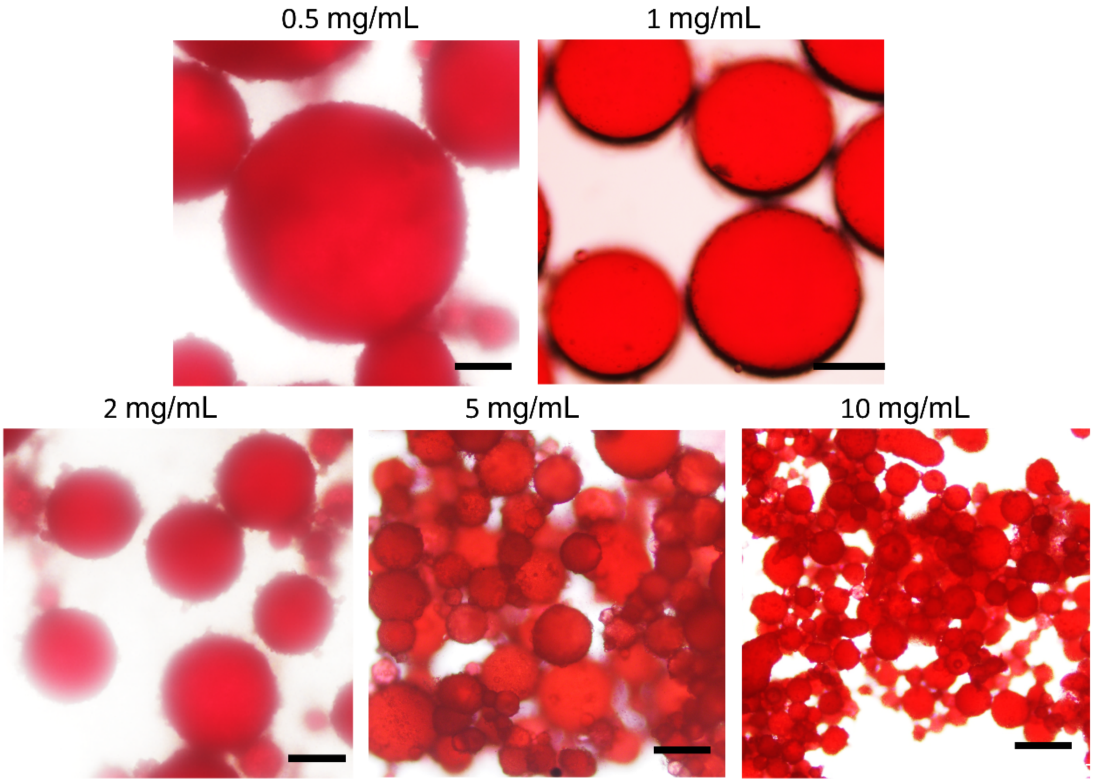

Supplementary Fig 4. Optical microscope images of the obtained Janus particle-engineered structural lipiodol droplets with different sizes fabricated by tuning the concentrations of Janus particles. Scale bars: the order from low concentration to high concentration is 100 μm, 100 μm, 100 μm, 100 μm, and 50 μm, respectively. Experiments were performed three times, with similar results.

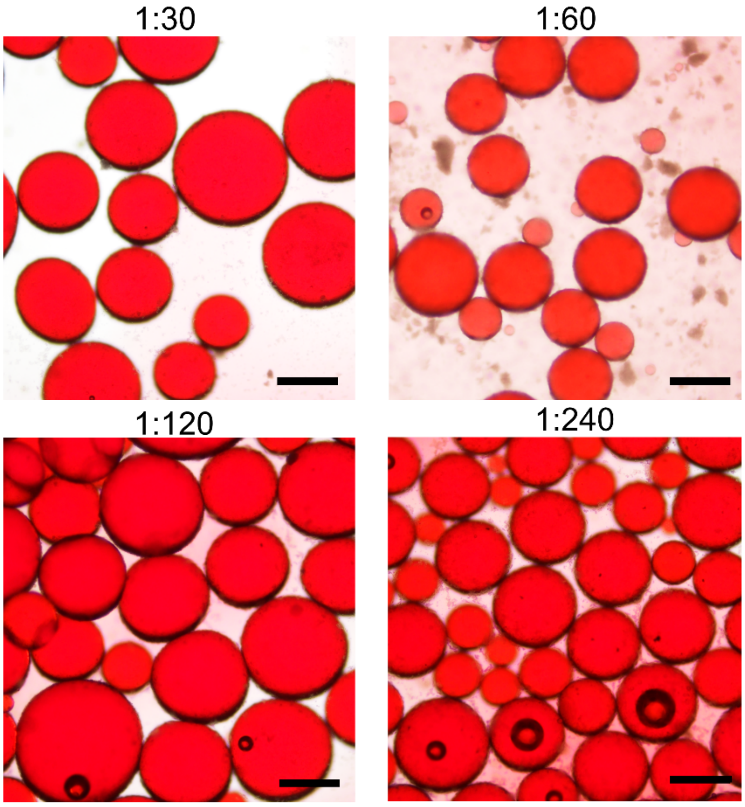

Supplementary Fig 5. Optical microscope images of the obtained Janus particle-engineered structural lipiodol droplets with different sizes fabricated by tuning the volume ratios of lipiodol and deionized water. Scale bars: the order from 1:30 to 1:240 is 500 μm, 500 μm, 100 μm, and 100 μm. Experiments were performed three times, with similar results.

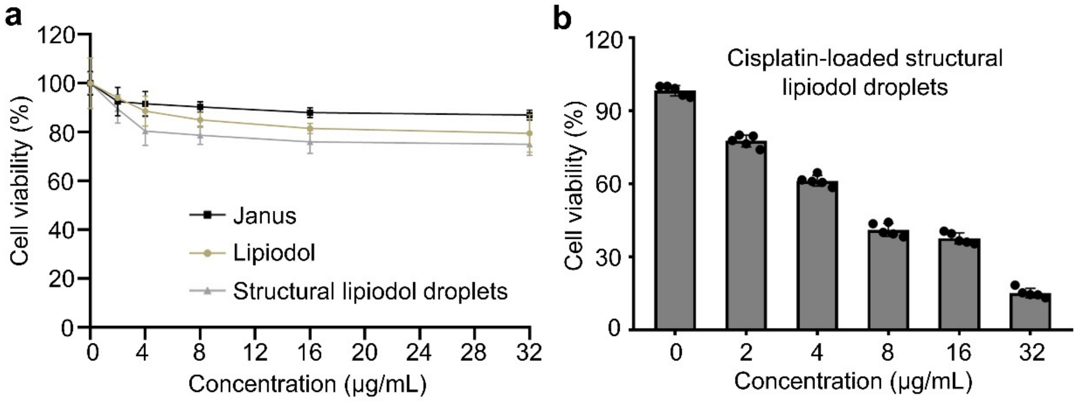

Supplementary Fig 6. Cytotoxicity of Janus particles, lipiodol, Janus particle-engineered structural lipiodol droplets and cisplatin-loaded Janus particle-engineered structural lipiodol droplets. (a) Cell viability of Janus particles, lipiodol, Janus particle-engineered structural lipiodol droplets in HUVEC cells. (b) Cell viability of cisplatin-loaded Janus particle-engineered structural lipiodol droplets in HepG2 cells. n = 5 biologically independent samples. Data are presented as means ± SD. Experiments were performed three times, with similar results.

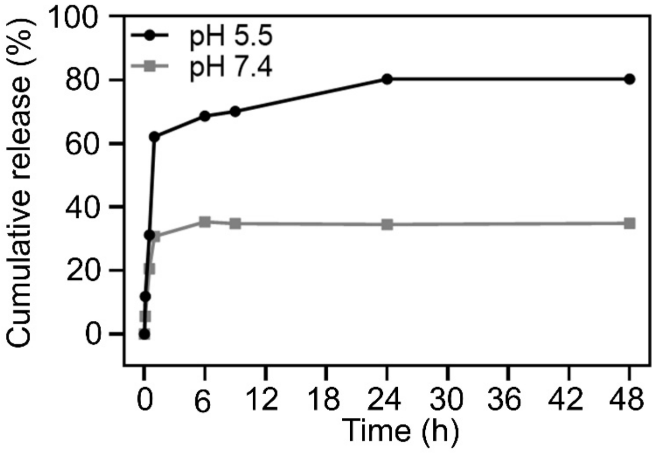

Supplementary Fig 7. The cumulative release of cisplatin within cisplatin-loaded Janus particle-engineered structural lipiodol droplets.

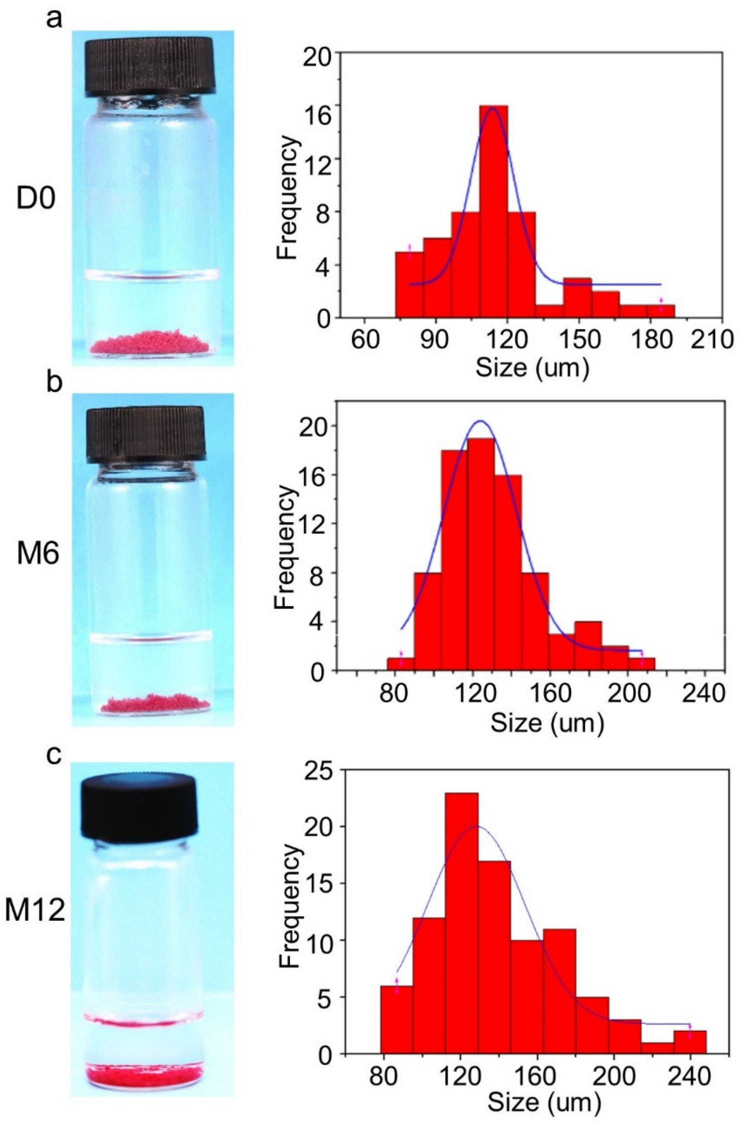

Supplementary Fig 8. Structural stability of the obtained Janus particle-engineered structural lipiodol droplets. (a) Photograph and size distributions of Janus particle-engineered structural lipiodol droplets at day 0. (b) Photograph and size distributions of Janus particle-engineered structural lipiodol droplets at 6 months. (c) Photograph and size distributions of Janus particle-engineered structural lipiodol droplets at 12 months.

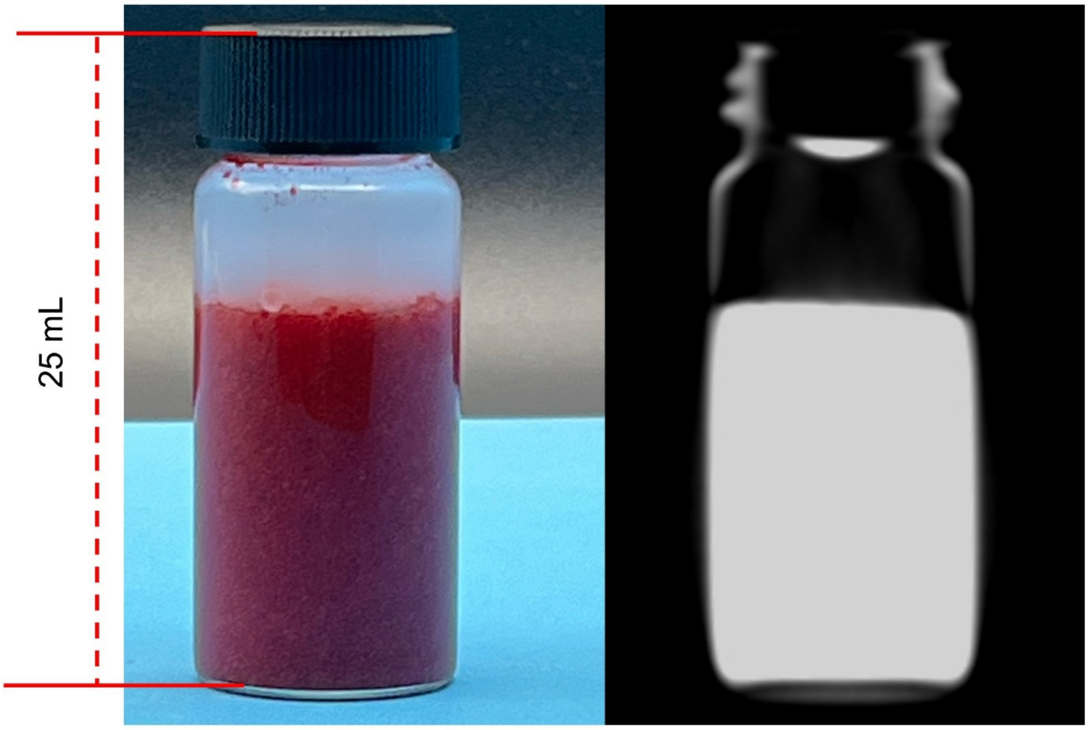

Supplementary Fig 9. Large-scale fabrication of Janus particle-engineered structural lipiodol droplets. Our approach enabled to produce approximately 25 g of Janus particle-engineered structural lipiodol droplets in one batch, suggesting a good potential for large-scale fabrication.

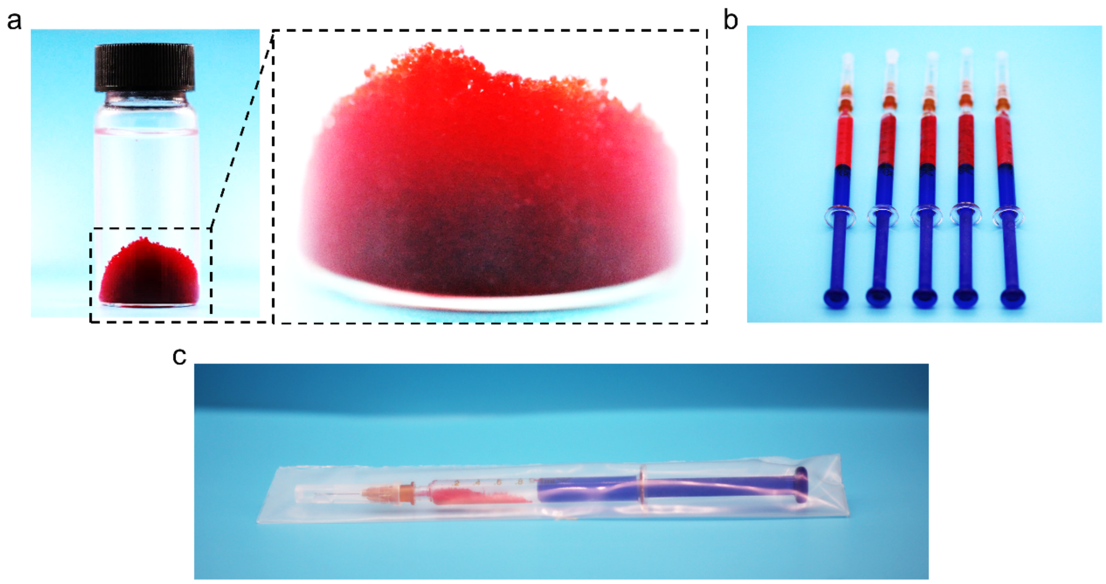

Supplementary Fig 10. The potential of Janus particle-engineered structural lipiodol droplets for application. (a-c) The Janus particle-engineered structural lipiodol droplets were easily packaged, indicating their good potential for future clinical application.

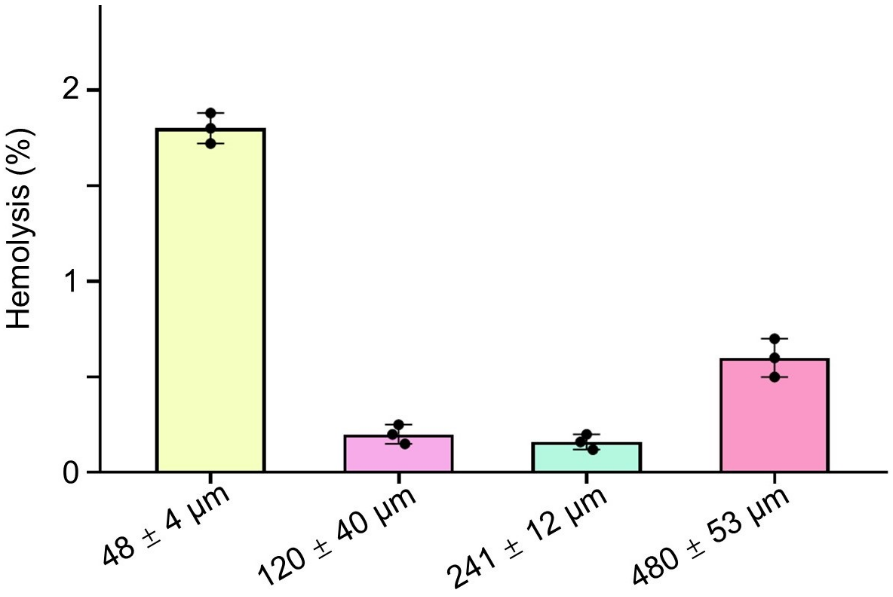

Supplementary Fig 11. Hemolysis rate of the Janus particle-engineered structural lipiodol droplets with different sizes. n = 3 biologically independent samples. Data are presented as means ± SD. Experiments were performed three times, with similar results.

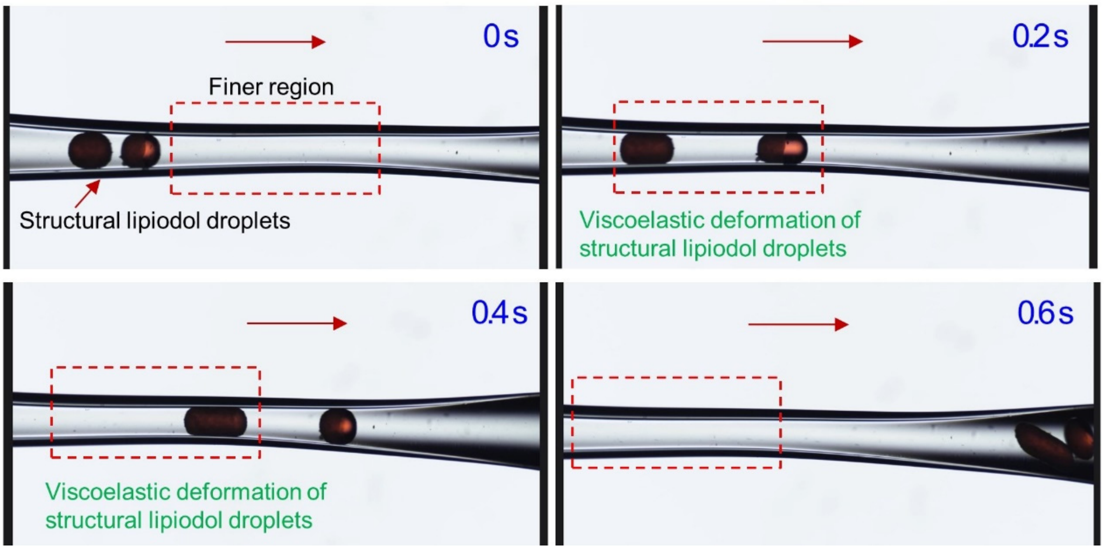

Supplementary Fig 12. The process of the Janus particle-engineered structural lipiodol droplets passed through the finer glass capillary tube.

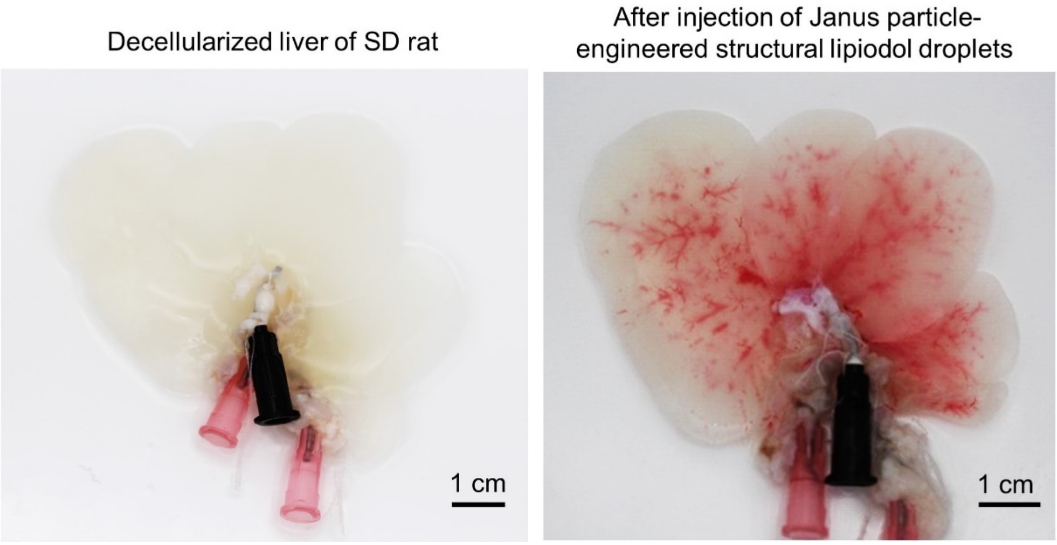

Supplementary Fig 13. Photograph of the decellularized liver of SD rat before and after injection of 0.5 mL of Janus particle-engineered structural lipiodol droplets. These Janus particle-engineered structural lipiodol droplets could be smoothly delivered into the blood vessels from arterial trunk to branch.

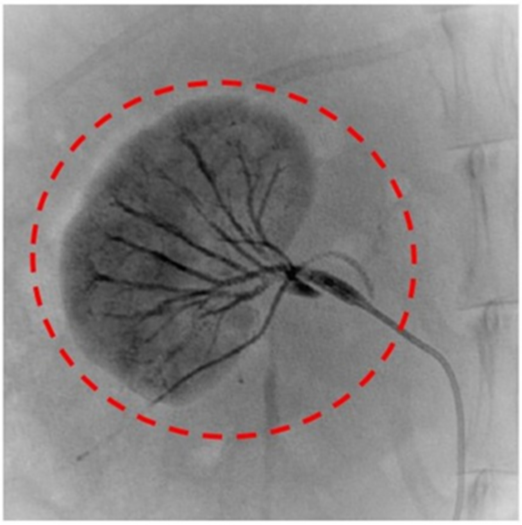

Supplementary Fig 14. Fluoroscopic detection of the distribution and intravascular embolization of the Janus particle-engineered structural lipiodol droplets. The Janus particle-engineered structural lipiodol droplets were efficiently deposited into blood vessels from renal artery to vascular branches.

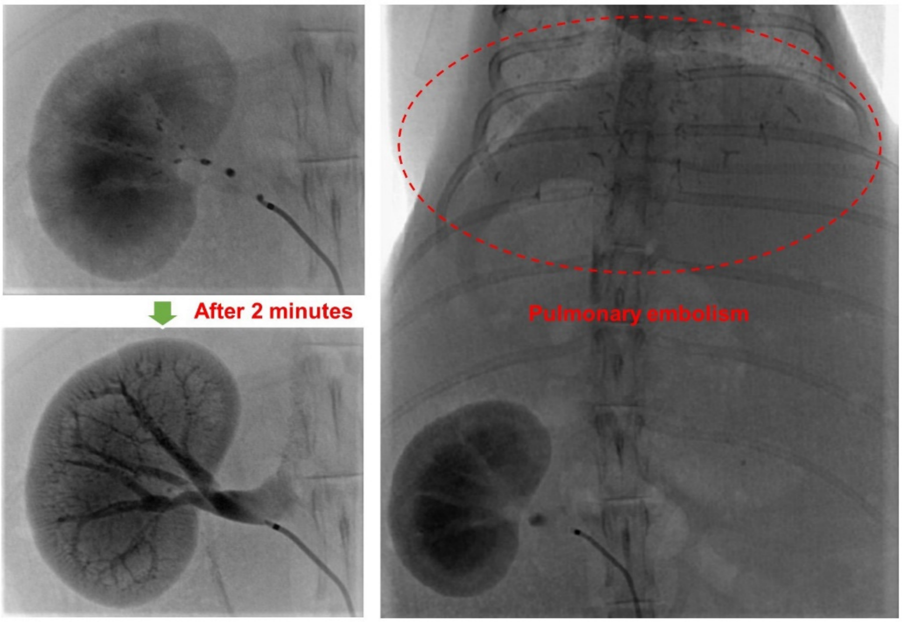

Supplementary Fig 15. The distribution and intravascular embolization of lipiodol-based emulsion. We observed that the some lipiodol-based emulsion would leak to renal vein after injection. Subsequently, the lipiodol-based emulsion could also leak into the lung, which might potentially risk pulmonary embolization.

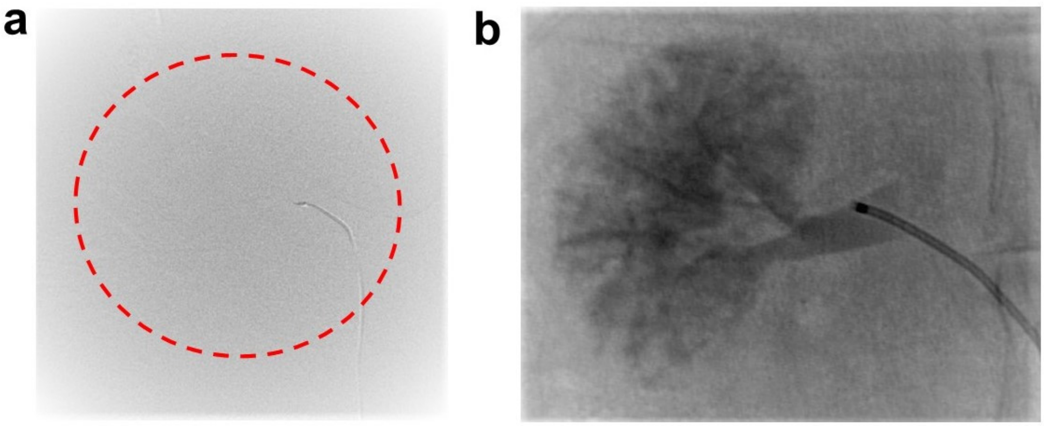

Supplementary Fig 16. The distribution and intravascular embolization of clinical 8spheres® beads in the blood vessels at day 0 post-embolization. (a) DSA images of clinical 8spheres® beads in the blood vessels on day 0 post-embolization. (b) Fluoroscopic detection of clinical 8spheres® beads in the blood vessels on day 0 post-embolization. The results indicated that the clinical 8sphere beads could not be visualized by DSA and fluoroscopy.

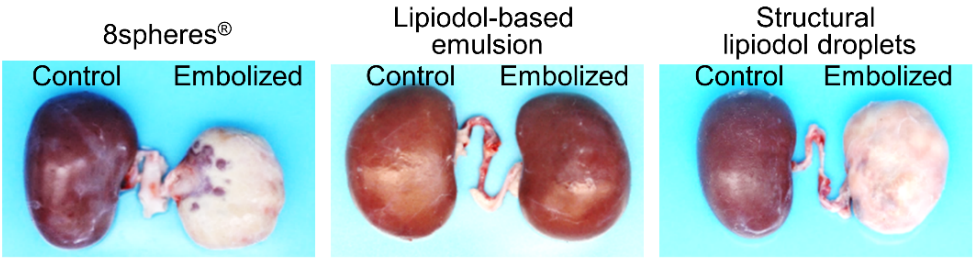

Supplementary Fig 17. Photograph images of excised kidneys. After 14 days post-embolization, the left and right kidneys were excised. Compared to the left kidney (Control), the embolized right kidney appeared milky white, and its volume was visibly reduced in the Groups of the Janus particle-engineered structural lipiodol droplets and 8spheres® beads.

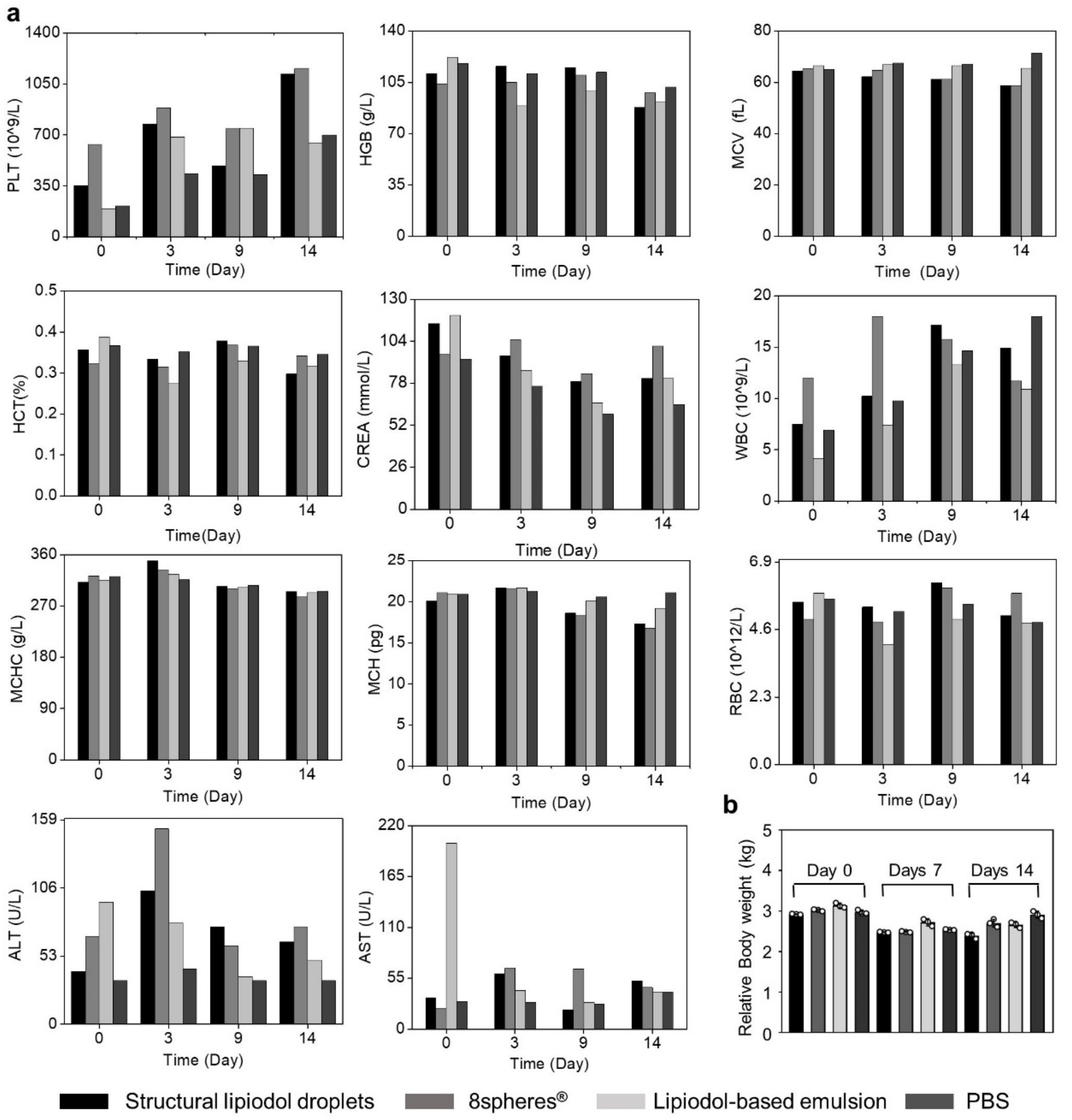

Supplementary Fig 18. The blood routine examination, liver and kidney function examination, blood coagulation examination, and weight test of Janus particle-engineered structural lipiodol droplets, clinical 8spheres® beads and Lipiodol-based emulsion after 14 days post-embolization. (a) Biochemical parameters of 8spheres® group, Janus particle-engineered structural lipiodol droplets group, Lipiodol-based emulsion group, and PBS group; (b) The weight of 8spheres® group, Janus particle-engineered structural lipiodol droplets group, lipiodol-based emulsion group, and PBS group. n = 3 biologically independent samples. Data are presented as means ± SD.

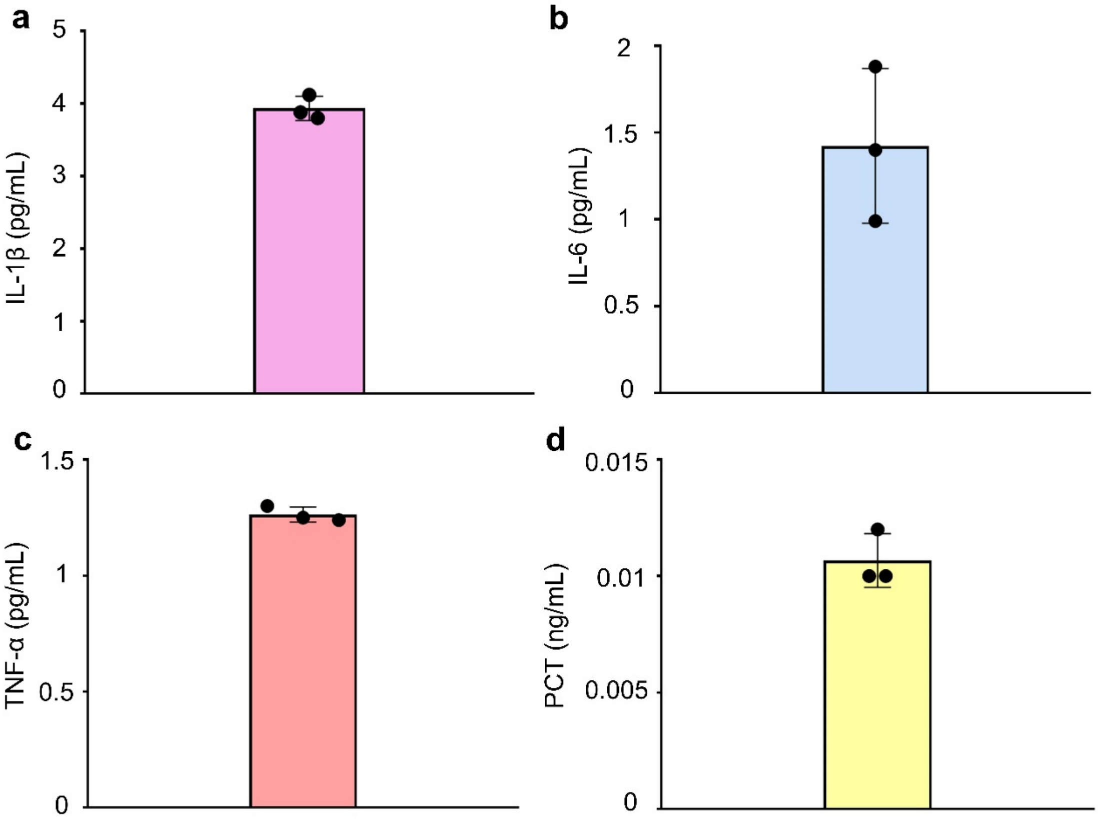

Supplementary Fig 19. The effect of Janus particle-engineered structural lipiodol droplets on sentinel pro-inflammatory in the blood of rabbit post-embolization. n = 3 biologically independent samples. Data are presented as means ± SD. Experiments were performed three times, with similar results.

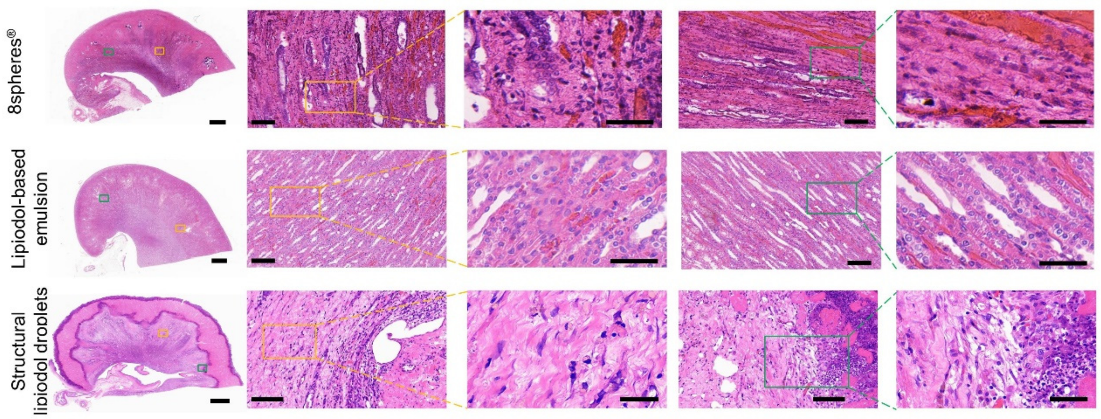

Supplementary Fig 20. H&E staining of the embolized right kidney post-embolization to look into inflammation response and fibrosis in the microenvironment. Scale bars are 2000 μm, 100 μm, 50 μm, 100 μm and 50 μm from left to right.

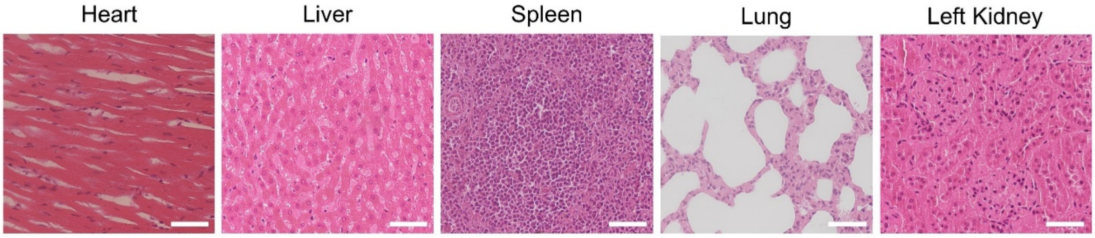

Supplementary Fig 21. H&E staining of major organs harvested from rabbit on days 45 post-embolization. Scale bars: 50 μm.

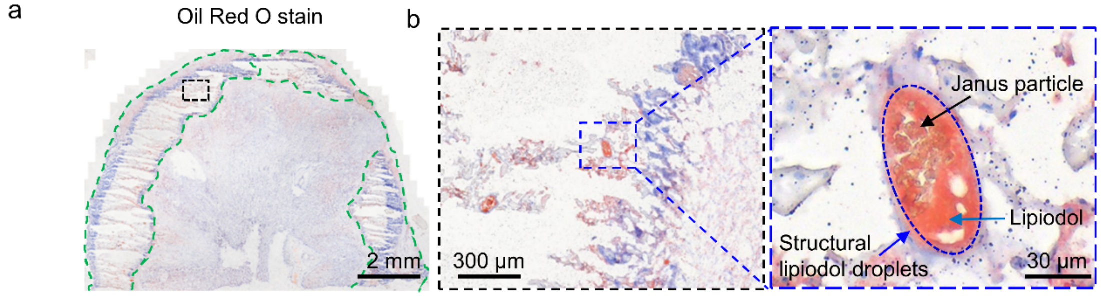

Supplementary Fig 22. Histological analysis of frozen sections of the embolizated right kidney in Janus particle-engineered structural lipiodol droplets group. The result showed that the renal arteries were effectively embolized by our Janus particle-engineered structural lipiodol droplets after 14 days post-embolization.

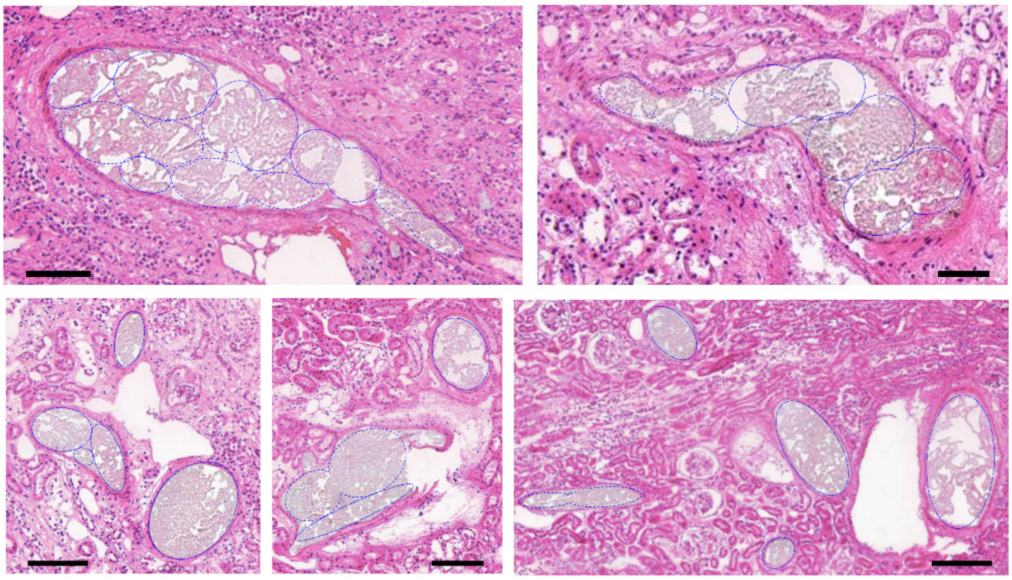

Supplementary Fig 23. H&E images of different levels of blood vessels in embolized right kidney by the Janus particle-engineered structural lipiodol droplets. Beyond the artery embolization, these Janus particle-engineered structural lipiodol droplets enabled to delivery to finer vasculature by viscoelastic deformation, into which their profiles of viscoelastic deformation could be clearly observed. Scale bars is 100 μm.

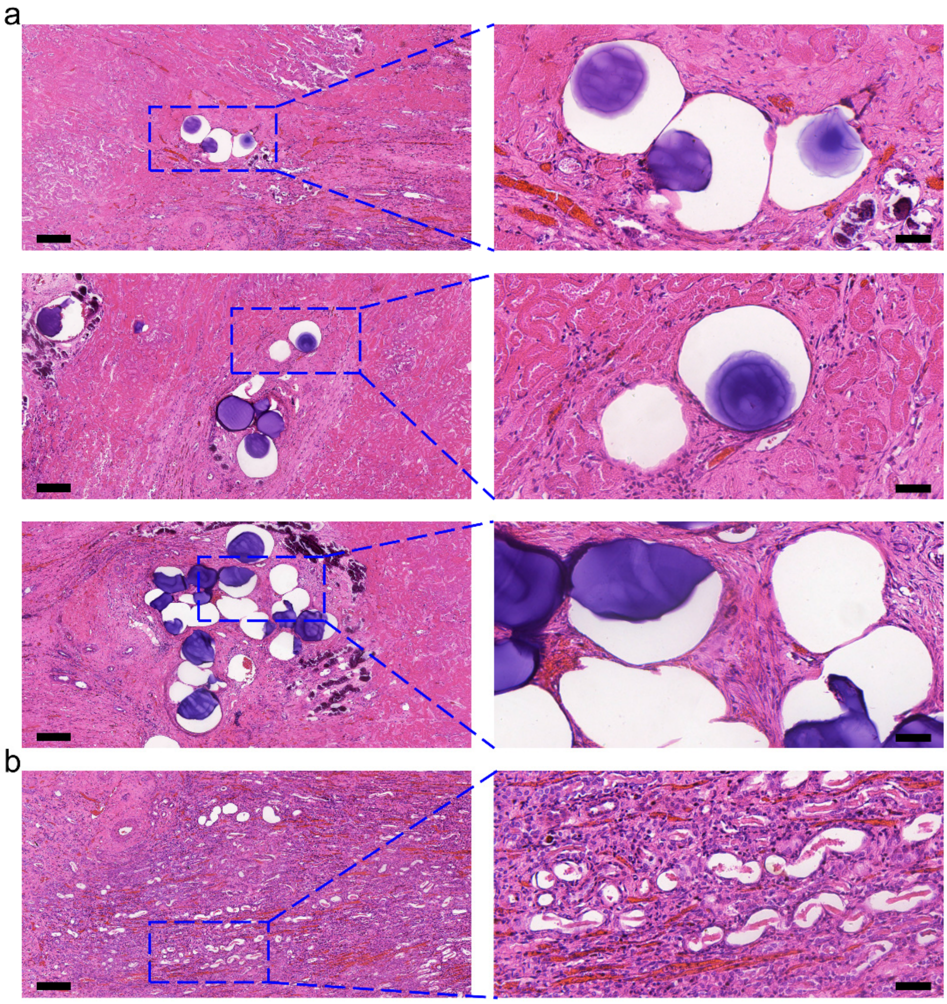

Supplementary Fig 24. H&E images of the different levels of blood vessels in embolized right kidney by the 8spheres® beads. (a) The distribution of clinical 8spheres® beads in the feeding artery. (b) And also, we did not observe the 8spheres® beads in the finer vasculatures. Scale bars: 100 μm on the left and 50 μm on the right.

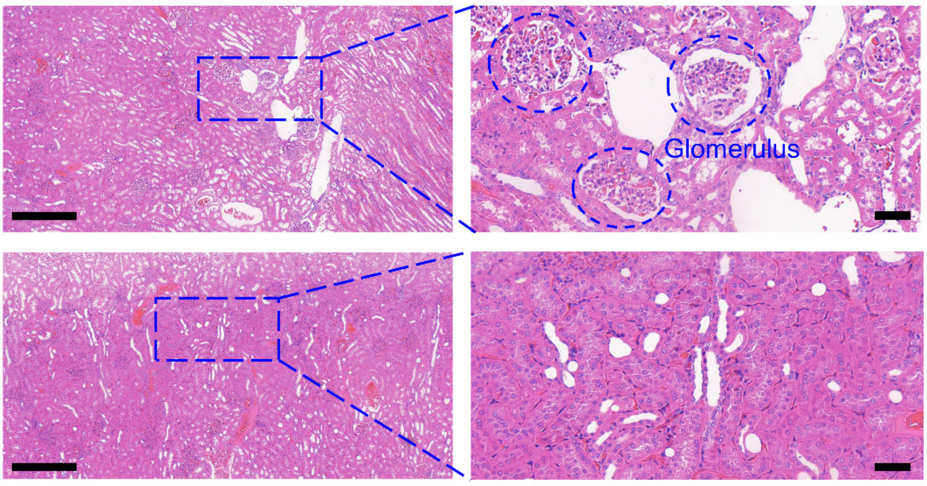

Supplementary Fig 25. H&E images of the different levels of blood vessels in embolized right kidney by the lipiodol-based emulsion. Scale bars: Left, 400 μm; Right, 50 μm.

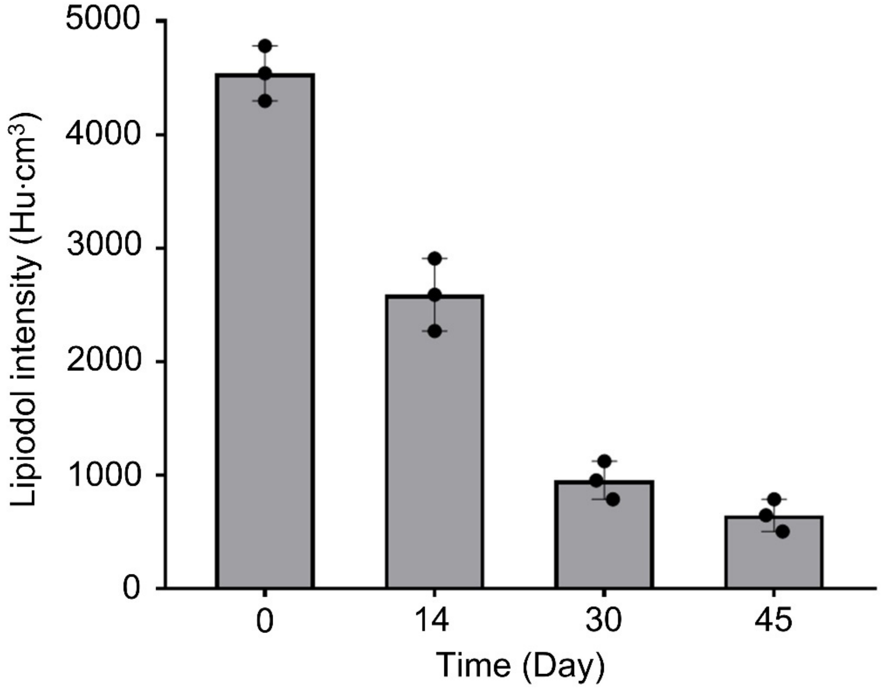

Supplementary Fig 26. The lipiodol intensity within Janus particle-engineered structural lipiodol droplets in rabbits at different time post-embolization. Accompanying with the volume reduction of kidney, the lipiodol within Janus particle-engineered structural lipiodol droplets could be metabolized gradually. Lipiodol intensity = deposition intensity of lipiodol in kidney × deposition volume of lipiodol in kidney. The deposition intensity of lipiodol was calculated by RadiAnt DICOM Viewer 2023.1 software, while the deposition volume of lipiodol was calculated by AW VolumeShare 4.7 (reformat, volume, GE Healthcare) software. $n = 3$ biologically independent samples. Data are presented as means ± SD. Experiments were performed three times, with similar results.

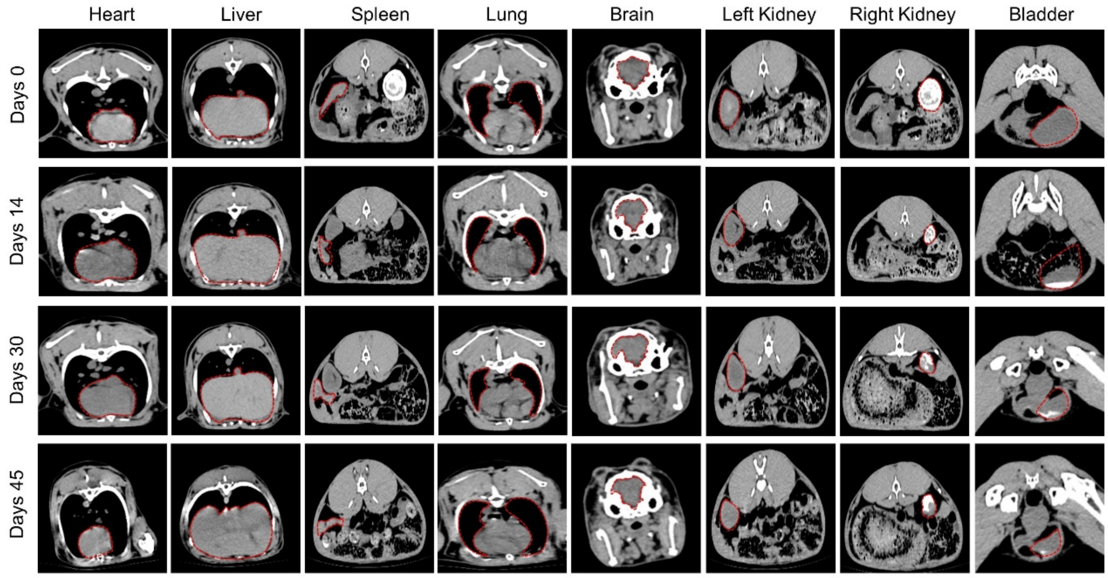

Supplementary Fig 27. CT images of the main organs at different times post-embolization. The results demonstrated that except for kidney and bladder, no evidence of lipiodol in the lung, liver, heart, spleen, brain, and normal left kidney, suggesting that lipiodol was mainly metabolized via kidney.

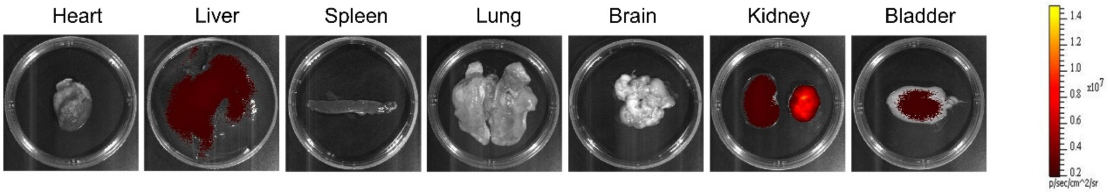

Supplementary Fig 28. The biodistributions of Janus particle-engineered structural lipiodol droplets on day 45 post-embolization. Rabbits were sacrificed on day 45 post-embolization and the main organs were carefully excised to observe the biodistributions of Janus particles. The result indicated that except for kidney, the fluorescence of Janus particles within the Janus particle-engineered structural lipiodol droplets was also observed in the liver and bladder on day 45 post-embolization. These results indicated the metabolization of Janus particles mainly proceeded with liver and kidney.

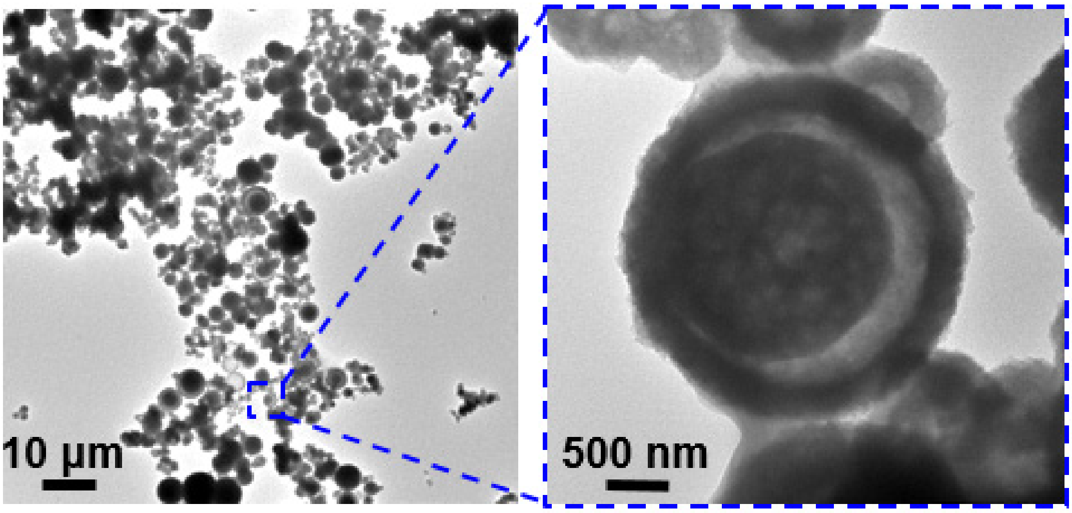

Supplementary Fig 29. TEM images of the Janus particles in the collected urine of rabbit post-embolization. In the collected urine of rabbits, we also clearly observed the Janus particles.

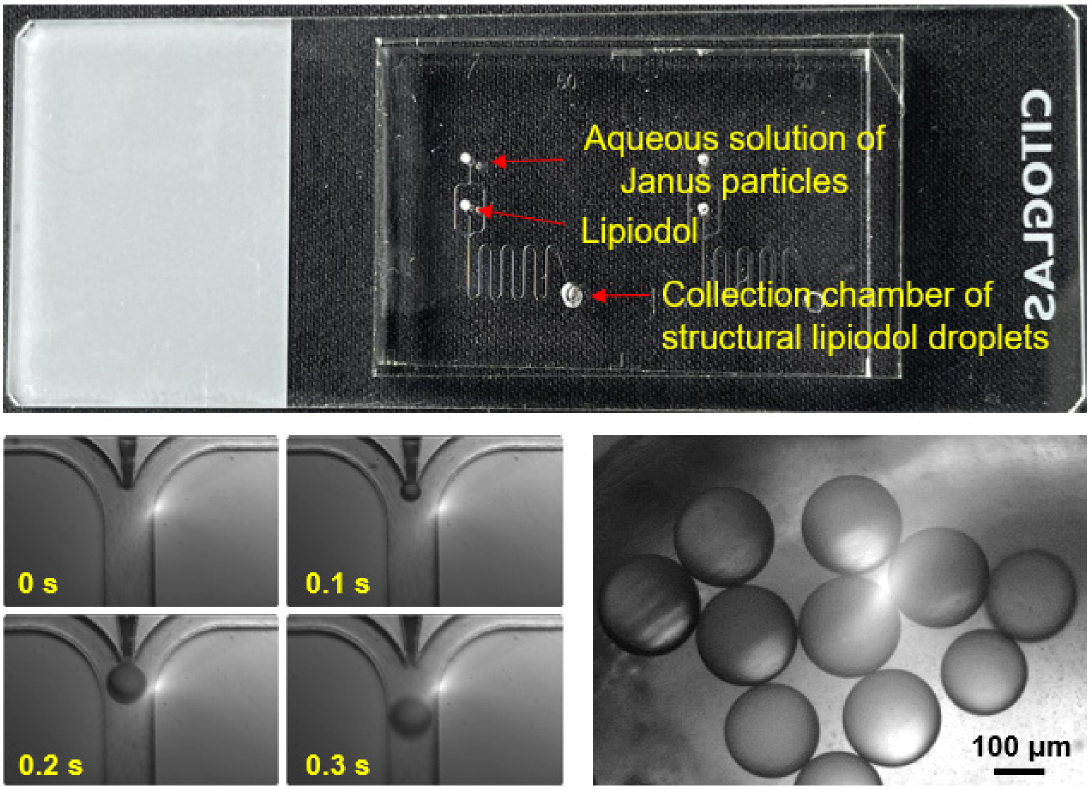

Supplementary Fig 30. Fabrication of uniform Janus particle-engineered structural lipiodol droplets by microfluidic technique. Experiments were performed three times, with similar results.

Supplementary Tab 1. Encapsulation efficiency of Janus particle-engineered structural lipiodol droplets containing cisplatin.

Cisplatin (mg)	3	6	10	20
Encapsulation efficiency (%)	38.5±0.12	93.5±0.23	90.11±0.11	45.32±0.03

Data were presented as means ± SD, n = 3.

Supplementary Movie.

Supplementary Movie 1. The smooth delivery of Janus particle-engineered structural lipiodol droplets in rabbit.

Supplementary Movie 2. The delivery of lipiodol-based emulsion in rabbit.
