# Supplementary material for: Janus particle-engineered structural lipiodol droplets for arterial embolization
Source: Nat Commun. 2023 Sep 11;14:5575. doi: 10.1038/s41467-023-41322-6 (PMC10495453; doi:10.1038/s41467-023-41322-6)
Supplement: Supplementary file 2 — Reporting Summary [file 41467_2023_41322_MOESM2_ESM.pdf]

nature portfolio

Corresponding author(s): Jun-Bing Fan

Last updated by author(s): Aug 15, 2023

Reporting Summary

Nature Portfolio wishes to improve the reproducibility of the work that we publish. This form provides structure for consistency and transparency in reporting. For further information on Nature Portfolio policies, see our Editorial Policies and the Editorial Policy Checklist.

Statistics

For all statistical analyses, confirm that the following items are present in the figure legend, table legend, main text, or Methods section.

n/a	Confirmed	
☐	☒	The exact sample size (n) for each experimental group/condition, given as a discrete number and unit of measurement
☐	☒	A statement on whether measurements were taken from distinct samples or whether the same sample was measured repeatedly
☐	☒	The statistical test(s) used AND whether they are one- or two-sided<br>*Only common tests should be described solely by name; describe more complex techniques in the Methods section.*
☒	☐	A description of all covariates tested
☒	☐	A description of any assumptions or corrections, such as tests of normality and adjustment for multiple comparisons
☐	☒	A full description of the statistical parameters including central tendency (e.g. means) or other basic estimates (e.g. regression coefficient) AND variation (e.g. standard deviation) or associated estimates of uncertainty (e.g. confidence intervals)
☐	☒	For null hypothesis testing, the test statistic (e.g. F, t, r) with confidence intervals, effect sizes, degrees of freedom and P value noted<br>*Give P values as exact values whenever suitable.*
☒	☐	For Bayesian analysis, information on the choice of priors and Markov chain Monte Carlo settings
☒	☐	For hierarchical and complex designs, identification of the appropriate level for tests and full reporting of outcomes
☒	☐	Estimates of effect sizes (e.g. Cohen's d, Pearson's r), indicating how they were calculated

Our web collection on statistics for biologists contains articles on many of the points above.

Software and code

Policy information about availability of computer code

Data collection	The optical images were visualized and collected using a Nikon DS Ri2 optical microscope. The confocal images visualized and collected using a Nikon AI confocal microscope. The SEM images were observed and collected under a Feiner Phenom Pharos electron microscope. The injectability of Janus particle-engineered structural lipiodol droplets through clinical catheters was tested using a mechanical tester (68TM-5, Instron). The DSA images were visualized and collected using a Philips machine. The CT images were observed and collected using a Siemens machine. The fluorescence imaging using an in vivo imaging system (IVIS Lumina II, Caliper, USA). Kidney volume datas were calculated by AW VolumeShare 4.7 software(GE Healthcare, USA).
Data analysis	All statistical analyses were analyzed by Microsoft office Excel 2019, Image J (v 2.0.0), Graphpad Prism (v 8.0) and Origin (v 8.5), Medical image data were analyzed using Radiant Dicom Viewer software package, Pathological data were analyzed using the Iviewer software package.

For manuscripts utilizing custom algorithms or software that are central to the research but not yet described in published literature, software must be made available to editors and reviewers. We strongly encourage code deposition in a community repository (e.g. GitHub). See the Nature Portfolio guidelines for submitting code & software for further information.

Data

Policy information about availability of data

All manuscripts must include a data availability statement. This statement should provide the following information, where applicable:

- Accession codes, unique identifiers, or web links for publicly available datasets
- A description of any restrictions on data availability
- For clinical datasets or third party data, please ensure that the statement adheres to our policy

The source data generated in this study are provided in the Supplementary Information/Source Data file. The full image dataset is available from the corresponding author upon request. All other data are available from the corresponding author upon request. Source data are provided with this paper

Research involving human participants, their data, or biological material

Policy information about studies with human participants or human data. See also policy information about sex, gender (identity/presentation), and sexual orientation and race, ethnicity and racism.

Reporting on sex and gender	No human researcher participants
Reporting on race, ethnicity, or other socially relevant groupings	No human researcher participants
Population characteristics	No human researcher participants
Recruitment	No human researcher participants
Ethics oversight	No human researcher participants

Note that full information on the approval of the study protocol must also be provided in the manuscript.

Field-specific reporting

Please select the one below that is the best fit for your research. If you are not sure, read the appropriate sections before making your selection.

☒ Life sciences ☐ Behavioural & social sciences ☐ Ecological, evolutionary & environmental sciences

For a reference copy of the document with all sections, see nature.com/documents/nr-reporting-summary-flat.pdf

Life sciences study design

All studies must disclose on these points even when the disclosure is negative.

Sample size	No statistical methods was used to predetermine the samples size. The sample sizes were determined as minimal to lower the cost and be sufficient to obtain statistically significant difference between experimental groups(n=3-5). For property measurement experiments, samples were prepared and tested at least twice. For in vivo studies, each group contains at least 3 for evaluating the statistical significance. These sample sizes also represent the standard practice for publication in this field and were described in figure legends. Each sample represents independent biological replicates.
Data exclusions	No data were excluded.
Replication	All experiments were repeated at least three times and all attempts at replication generated similar results.
Randomization	The samples were randomly grouped.
Blinding	Blinding was not required in this study and investigators were not blinded to group allocation as all data in this study were analyzed equivalently within defined experimental groups.

Reporting for specific materials, systems and methods

We require information from authors about some types of materials, experimental systems and methods used in many studies. Here, indicate whether each material, system or method listed is relevant to your study. If you are not sure if a list item applies to your research, read the appropriate section before selecting a response.

Materials & experimental systems

n/a	Involved in the study
☒	☐ Antibodies
☐	☒ Eukaryotic cell lines
☒	☐ Palaeontology and archaeology
☐	☒ Animals and other organisms
☒	☐ Clinical data
☒	☐ Dual use research of concern
☒	☐ Plants

Methods

n/a	Involved in the study
☒	☐ ChIP-seq
☒	☐ Flow cytometry
☒	☐ MRI-based neuroimaging

Eukaryotic cell lines

Policy information about cell lines and Sex and Gender in Research

Cell line source(s)	Human umbilical vein endothelia cells (HUVEC) cells were obtained from the Cell Bank of Shanghai institute of Cell Biology, Chinese Academy of Sciences (Shanghai China). Hepatocellular carcinoma (HepG2) cells were kindly provided by Dr. Yi Gao from The Second Clinical Medical College of Southern Medical University.
Authentication	The cell line we used was morphologically confirmed according to the information provided by SIBS.
Mycoplasma contamination	All cells were tested to be free of mycoplasma contamination.
Commonly misidentified lines (See ICLAC register)	No cell lines used are listed in the database of commonly misidentified lines.

Animals and other research organisms

Policy information about studies involving animals; ARRIVE guidelines recommended for reporting animal research, and Sex and Gender in Research

Laboratory animals	New Zealand white rabbits(9-10 months old, 3.0-3.5 kg) and SD rats (2-3 months, 200-250 g) were obtained from the Laboratory Animal Center of Southern Medical University. Temperature 25±1℃, humidity at 40–70%, dark/light cycle 6 pm-6 am.
Wild animals	No wild animal data generated used in the study.
Reporting on sex	The animal data generated here was analyzed not considering sex as avarible.
Field-collected samples	No field-collected samples were used in the study.
Ethics oversight	All animal testing procedures were reviewed and approved by the Animal Care and Use Committee of Southern Medical University (permit number: NFYY-2021-0847). All animal experiments were conducted in accordance with the regulation of Chian and Nangfang hospital on the health and welfare of laboratory animals.

Note that full information on the approval of the study protocol must also be provided in the manuscript.
