# Supplementary material for: Janus particle-engineered structural lipiodol droplets for arterial embolization
Source: Nat Commun. 2023 Sep 11;14:5575. doi: 10.1038/s41467-023-41322-6 (PMC10495453; doi:10.1038/s41467-023-41322-6)
Supplement: Supplementary file 3 — Description of Additional Supplementary Files [file 41467_2023_41322_MOESM3_ESM.pdf]

Title: Supplementary Movie 1.
Description: The smooth delivery of Janus particle-engineered structural lipiodol droplets in rabbit.

Title: Supplementary Movie 2.
Description: The delivery of lipiodol-based emulsion in rabbit.
